# Dietary Acid Load and Potassium Intake Associate with Blood Pressure and Hypertension Prevalence in a Representative Sample of the German Adult Population

**DOI:** 10.3390/nu10010103

**Published:** 2018-01-19

**Authors:** Danika Krupp, Jonas Esche, Gert Bernardus Maria Mensink, Stefanie Klenow, Michael Thamm, Thomas Remer

**Affiliations:** 1 DONALD Study Dortmund, Department of Nutrition and Food Sciences, Nutritional Epidemiology, University of Bonn, 44225 Dortmund, Germany; jesche@uni-bonn.de (J.E.); remer@uni-bonn.de (T.R.); 2 Robert Koch-Institute, Department of Epidemiology and Health Monitoring, Robert Koch Institute, 13302 Berlin, Germany; MensinkG@rki.de (G.B.M.M.); KlenowS@rki.de (S.K.); ThammM@rki.de (M.T.)

**Keywords:** dietary acid load, PRAL, potassium intake, blood pressure, hypertension, DEGS1

## Abstract

Diets rich in fruits and vegetables, like the Dietary Approaches to Stop Hypertension (DASH)-diet, are usually characterized by high potassium intake and reduced dietary acid load, and have been shown to reduce blood pressure (BP). However, the relevance of potential renal acid load (PRAL) for BP has not been compared with the relevance to BP of urinary biomarker (K-urine)- and dietary food frequency questionnaire (K-FFQ)-based estimates of potassium intake in a general adult population sample. For 6788 participants (aged 18–79 years) of the representative German Health-Interview and Examination Survey for Adults (DEGS1), associations of PRAL, K-urine, and K-FFQ with BP and hypertension prevalence were cross-sectionally examined in multivariable linear and logistic regression models. PRAL was significantly associated with higher systolic BP (*p* = 0.0002) and higher hypertension prevalence (Odds ratio [OR] high vs. low PRAL = 1.45, *p* = 0.0004) in models adjusted for age, sex, body mass index (BMI), estimated sodium intake, kidney function, relevant medication, and further important covariates. Higher estimates of K-FFQ and K-urine were related to lower systolic BP (*p* = 0.04 and *p* < 0.0001) and lower hypertension prevalence (OR = 0.82, *p* = 0.04 and OR = 0.77, *p* = 0.02) as well as a lower diastolic BP (*p* = 0.03 and *p* = 0.0003). Our results show, for the first time in a comparative analysis of a large representative population sample, significant relationships of BP and hypertension prevalence with questionnaire- and biomarker-based estimates of potassium intake and with an estimate of dietary acid load.

## 1. Introduction

Current guidelines on the management of arterial hypertension recommend lifestyle changes including dietary measures to prevent the development of high blood pressure (BP) and to assist in reducing BP as well as cardiovascular disease (CVD) risk in hypertensives [1]. Apart from a reduction in salt and alcohol intake, increases in the consumption of fruits and vegetables, and vegetarian diets as well as Dietary Approaches to Stop Hypertension (DASH)-type diets have been shown to reduce BP in interventional and observational studies [2,3,4]. Of note, a recent review and meta-analysis of dietary interventions for BP-reduction indicated that among common dietary interventions (including low-sodium diets), the DASH-type diet may be the most effective [4]. Different aspects of the above-mentioned dietary patterns may account for the observed BP decreases. Apart from increased intakes of several minerals including potassium, for which substantial evidence for a BP-reducing effect exists [5,6], a lowered nutritive proton load is a common characteristic of diets rich in fruits and vegetables, including DASH-type diets. Main determinants of the daily dietary acid load include high intakes of protein as well as phosphorus as acid-producing components, whereas high intakes of fruits, vegetables, and potatoes reduce the daily proton load. The potential renal acid load (PRAL) is an established marker of the diet-dependent proton load and has been used in several studies on different health outcomes in adults and children [7,8,9]. Regarding the potential relationship of dietary acid load with BP, most [10,11,12,13] but not all [14,15] observational studies conducted in recent years suggest a corresponding direct link. Increases in dietary proton load have also been shown to induce changes in systemic acid–base status [16,17] and different markers of such subclinical forms of metabolic acidosis have been related to BP and hypertension incidence as well [18,19,20]. With respect to available mechanistic evidence, several animal models have linked disturbances in acid–base balance to (salt-sensitive) hypertension [21,22], and these disturbances may already be present before the onset of hypertension [21]. Also in humans, salt sensitivity of BP was associated with lower arterial pH [23].

To elaborate on the potential BP-reducing mechanisms of plant-based diets, the aim of the current analysis was to assess the relation between diet-dependent acid load and BP as well as hypertension prevalence in a sample of the general adult population living in Germany, and to compare this association with the (established) relevance of potassium intake to BP, concurrently considering the possible confounding effects of sodium intake, kidney function, and several further risk factors for hypertension.

## 2. Materials and Methods

### 2.1. Study Population

Data for the present analysis came from the first wave of the German Health Interview and Examination Survey for Adults (“Studie zur Gesundheit Erwachsener in Deutschland”, DEGS1), which was conducted between 2008 and 2011. Details on the concept and design of DEGS, a nationally representative study which is part of the health monitoring system at the Robert Koch-Institute (RKI), Berlin, have been previously described [24]. In brief, during the first examination wave, persons aged 18–79 years living in Germany were randomly selected according to a nationwide two-stage clustered sample design and examined at one of the 180 study centers, resulting in a net sample of 7115 participants. Of these, *n* = 2923 were revisiting participants of the German National Health Interview and Examination Survey 1998 (GNHIES98). The study was approved by the ethical committee of Charité University Medicine, Berlin (No. EA2/047/08) and conducted according to guidelines provided by the Federal and State Commissioners for Data Protection. Informed written consent was obtained from all participants. For the present analysis, *n* = 6788 participants with complete information on BP and body mass index (BMI), serum and urine samples for laboratory analyses as well as dietary data were selected. Of these, *n* = 6765 also had complete information on hypertensive status.

### 2.2. Dietary Intake

A validated [25] semi-quantitative, self-administered food frequency questionnaire (FFQ), inquiring about consumption frequencies and portion sizes of 53 food groups during the preceding 4 weeks, was used to assess dietary intake in DEGS1. Dietary acid load was determined by calculating the potential renal acid load (PRAL), i.e., a marker of the dietary impact on human acid–base status [26] as:

PRAL (mEq/day) = 0.49 × protein (g/day)
+0.037 × phosphorus (mg/day)
−0.021 × potassium (mg/day)
−0.026 × magnesium (mg/day)
−0.013 × calcium (mg/day).

To estimate the individual daily acid load, PRAL values were assigned to the food groups of the DEGS1-FFQ. In a first step, PRAL values of relevant single foods of the respective food groups were calculated using nutrient information on individual food items from the ‘German Food Content and Nutrition Data Base’ (Bundeslebensmittelschlüssel (BLS)), version 3.02 [27]. In a second step, additional data from the ‘National Nutrition Survey (NVS) II’ [28] were used to obtain more detailed information on the distribution of specific single food consumption in the German population regarding the rather broad DEGS1 food groups. Subsequently, single food PRAL values, weighed according to the NVS II distribution, were used to obtain DEGS1 food group-specific PRAL estimates (mEq/100 g) for individual PRAL calculation. Alcohol intake was quantified by summing the alcohol content of consumed beer, non-alcoholic beer (still containing minor amounts of alcohol), wine, spirits and liquor, in order to categorize participants as non-drinkers (0 g/day), light drinkers (men: >0 to 20 g/day, women: >0 to 10 g/day) or heavy drinkers (men: >20 g/day, women >10 g/day) [29]. An index of potassium intake (mg/day) was derived from FFQ food group consumption and weighted average nutrient contents obtained from the German Nutrition Survey 1998 [30].

### 2.3. Measurements and Laboratory Analyses

BP was measured according to standardized procedures with an automated oscillometric device (Datascope Accutorr Plus, Mahwah, NJ, USA). For each participant, three consecutive BP measurements were taken at 3-min intervals after an initial 5-min rest and following a non-strenuous part of the examination. During the measurements, the participant’s back was supported in upright position, with the right forearm resting on a table at heart level, and the legs uncrossed with feet on the floor. Three different cuff sizes were used depending on the right mid-upper arm circumference. Height and weight were measured with standardized procedures in lightly clothed participants and were used to calculate BMI as weight in kilograms divided by height in meter squared. Venous blood was drawn using Vacutainer EDTA and gel tubes (Becton Dickinson, Franklin Lakes, NJ, USA) and was immediately centrifuged and separated. Fasting duration and time of blood sampling was documented and aliquots of serum samples were stored at −40 °C within one hour. For storage and detailed analysis, serum and whole blood samples were brought to the central epidemiology laboratory unit at the RKI, Berlin. Analyses were performed on the Architect platform CI 8200 (Abbott, Chicago, IL, USA), with enzymatic methods used for determination of total cholesterol (CHOD-PAP) and glucose (hexokinase/G-6-PDH). A colorimetric method (picrate) was used to measure concentrations of serum and urinary creatinine. Glomerular filtration rate (eGFR) was estimated from serum creatinine using the four-variable Modification of Diet in Renal Disease (MDRD) Study equation [31]. Potassium and sodium concentrations in spot urine samples were determined with ion-sensitive electrode (ISE; indirect method) and subsequently used for estimation of 24-h excretion rates from urinary mineral–creatinine ratios according to a recently published prediction equation [32]. Urinary albumin excretion was determined with semi-quantitative test strips (Micral, Roche Diagnostics, Grenzach-Wyhlen, Germany). 

### 2.4. Other Variables

Physical activity was assessed with standardized questionnaires and was classified into ‘no sports activity’, ‘<2 h sports per week’, or ‘≥2 h sports per week’. Reported information on smoking frequency and number of cigarettes consumed per day was used to categorize subjects into (occasional) smokers, ex-smokers and non-smokers. A three-stage index of socioeconomic status (low, middle, high) was derived from questionnaire information on education, occupation and household income as has been previously reported in detail [33]. Information on current medication (prescription or over-the-counter) taken in the previous 7 days was verified in a computer-assisted personal medication interview and by barcode scanning of original drug packages brought to the study center. Antihypertensive medication was defined as use of antihypertensive drugs (C02), diuretics (C03), beta-blockers (C07), calcium channel blockers (C08) or angiotensin-converting enzyme (ACE) inhibitors (C09) according to the Anatomical Therapeutic Chemical (ATC) classification system. A number of previous physician-diagnosed diseases were assessed in a standardized personal interview.

### 2.5. Statistical Analyses

Statistical analysis system (SAS, version 9.2, SAS Institute, Cary, NC, USA) was used for statistical analyses and a *p*-value < 0.05 was considered significant in all statistical tests. To take account of deviations from the population structure in Germany as of 31 December 2010, a weighting factor considering age, sex, education, nationality, region, and community type was used. With respect to former GNHIES98-participants, the weighting factor also considered re-participation probabilities. Survey procedures in SAS for complex samples were used to account for the weighting and clustering in DEGS1 due to the two-stage sampling procedure. Mean values of the second and third measurement of systolic and diastolic BP were used in all analyses. Hypertension was defined as treatment with ATC-coded antihypertensive medication in those reporting a diagnosis of hypertension or as systolic BP ≥140 mmHg or diastolic BP ≥90 mmHg.

Descriptive data are presented in sex-balanced quintiles of dietary PRAL. Quintile construction was based on sex-specific PRAL distributions and the respective quintiles for males and females were combined. Differences in characteristics between the quintiles were tested with ANOVA and Kruskal–Wallis tests for normally and non-normally distributed continuous variables, respectively, and with Chi-square tests in the case of categorical variables. For assessment of the association between dietary (or urinary) predictors and BP, median values for the respective predictors were calculated for sex-balanced quintiles and used as continuous variables in the regression analyses. In basic linear regression models, adjustment was made for sex, age and BMI as known important predictors of BP. Adjusted models further considered relevant behavioral and dietary characteristics, cardiovascular risk factors, renal function, and medication. Among the biologically plausible potential confounders, only those were included in the final models that either modified the β-coefficient of the main predictor by >10% or were independently associated with BP. The latter was the case for all finally included covariates (see footnote Table 2). Additionally tested covariates, such as “physician-diagnosed diabetes” and “physician-diagnosed dyslipidemia” did not meet the inclusion criteria. Confounder selection was based on the basic linear regression model for PRAL and systolic BP and the same adjustment was used in all final models for reasons of comparability. Sensitivity analyses were performed in *n* = 4677 participants not on antihypertensive medication as well as in *n* = 5873 participants with apparently normal kidney function defined as an eGFR > 60 mL/min/1.73 m^2^, no significant microalbuminuria (<50 mg/L albumin on a semiquantitative test strip), and no physician-diagnosed kidney function impairment. Multiple logistic regression models with the same adjustment as used for the linear regression analyses (except for antihypertensive medication use) were used to evaluate the association of PRAL and potassium with hypertension prevalence. In these analyses, predictors were categorized into sex-balanced tertiles (T1: low, T2: middle, T3: high) and adjusted odds ratios (ORs) of hypertension were presented for high (T3) versus low (T1) values of the respective predictor. 

## 3. Results

### 3.1. Descriptive Data

Characteristics of the study population across PRAL quintiles are presented in Table 1. Median PRAL values ranged from −30.8 mEq/day to +15.5 mEq/day. Due to the sex-specific quintile categorization, the percentage of women did not differ significantly between the quintiles. Regarding age and BMI, lowest values were observed in the highest PRAL quintile, while no clear trend was discernible for systolic and diastolic BP. In contrast, hypertension prevalence differed significantly between the PRAL quintiles, ranging from 36.3% in the second quintile to 24.6% in the fifth quintile, with use of diuretics and β-blockers following a similar pattern. Mean values of serum cholesterol were lowest and eGFR was highest in participants with the highest PRAL. Most behavioral characteristics except for participation in sports activity also differed significantly across the PRAL quintiles. With respect to dietary intakes, participants with the lowest PRAL values had the highest intakes of potassium and milk products as well as fruits and vegetables, while participants in the highest PRAL quintile consumed significantly more meat and meat products. In contrast, estimated salt intake was not different across the PRAL range. 

### 3.2. Linear Regression

In basic linear regression models adjusted for age, sex, and BMI, higher PRAL values were significantly related to higher systolic BP (β = 0.049, *p* = 0.0005) but not to diastolic BP (β = 0.012, *p* = 0.2) (Table 2). Inverse but non-significant associations with systolic and diastolic BP were observed for FFQ-derived potassium intake in the basic models (β = −0.333, *p* = 0.08 and β = −0.173, *p* = 0.2 for systolic and diastolic BP, respectively), whereas for estimated 24-h potassium excretion, a significant inverse association with systolic BP (β = −0.012, *p* = 0.04) was discernible. For diastolic BP, the relationship with potassium excretion was of only marginal significance (β = −0.007, *p* = 0.06) in basic models. Additional adjustment for the size of BP cuff, fasting duration, cardiovascular risk factors (serum glucose and total cholesterol), eGFR, behavioral factors (smoking status, alcohol consumption), medication (diuretics and β-blockers), and estimated 24-h sodium excretion changed the PRAL–BP associations only marginally (β = 0.052, *p* = 0.0002 and β = 0.015, *p* = 0.1 for systolic and diastolic BP, respectively; adjusted models, Table 2). Regarding the FFQ-based potassium intake, significant inverse associations emerged for systolic (β = −0.397, *p* = 0.04) as well as diastolic BP (β = −0.255, *p* = 0.03) in adjusted models. Similarly, associations of potassium excretion with systolic (β = −0.033, *p* < 0.0001) and diastolic BP (β = −0.015, *p* = 0.0003) were strengthened upon confounder adjustment.

In sensitivity analysis in the subgroup of *n* = 4677 participants not taking antihypertensive medication (Table 3), associations were slightly weakened but remained significant for PRAL with systolic BP (*p* = 0.01) and for potassium excretion with systolic (*p* = 0.0001) and diastolic BP (*p* = 0.003) in adjusted models. In contrast, FFQ-derived potassium intake was not a significant predictor of BP in the subgroup of untreated DEGS1-participants in basic or adjusted models.

### 3.3. Logistic Regression

**Figure 1:**
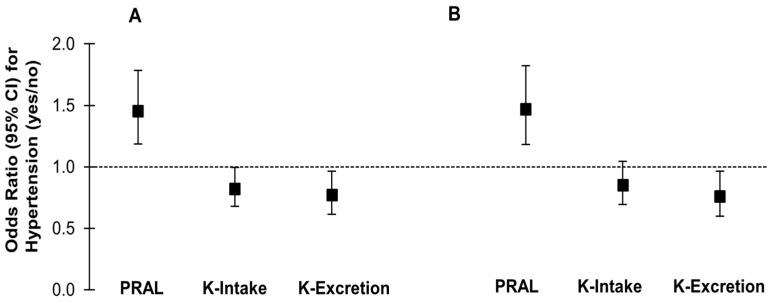
Odds ratios (95% CI) for hypertension (yes/no) comparing the highest (T3) with lowest (T1) tertiles of potential renal acid load (PRAL), food frequency questionnaire-derived potassium intake (K-Intake), and potassium excretion in the total study sample (6765) (**A**) and in a subsample (*n* = 5854) (**B**) excluding participants with impaired renal function. Odds ratios were calculated using logistic regression models adjusted for age, sex, and body mass index, size of blood pressure cuff, fasting duration (> or <8 h), smoking status, natrium excretion, alcohol intake, estimated glomerular filtration rate, serum glucose, and total cholesterol. CI, confidence interval.

With respect to hypertension prevalence (Figure 1A), logistic regression models demonstrated higher odds of hypertension for the highest tertile of dietary PRAL (OR (T3 vs. T1): 1.45, *p* = 0.0004) compared to the lowest PRAL tertile as the reference category in the adjusted model. Regarding FFQ-derived potassium intake, the respective OR of hypertension prevalence for high (T3) vs. low (T1) intake was 0.82 (*p* = 0.04). Estimated 24-h potassium excretion was a significant predictor of hypertension prevalence in the adjusted model as well (OR (T3 vs. T1): 0.77, *p* = 0.02). Excluding participants with impaired renal function resulted in very similar results except for FFQ-derived potassium intake, for which the difference in hypertension prevalence between low and high intakes was no longer significant (OR (T3 vs. T1): 0.85, *p* = 0.1) (Figure 1B).

## 4. Discussion

In our cross-sectional analyses in a comparably large representative sample of the general adult population living in Germany, we demonstrated that higher PRAL values, indicative of a higher diet-dependent proton load, are related to higher systolic BP and hypertension prevalence independent of estimated 24-h sodium excretion, BMI, eGFR, and several further established risk factors for elevated BP. Results were also confirmed in the subgroup not receiving antihypertensive treatment and in those participants with apparently normal kidney function. These findings are in line with several recent observational studies demonstrating similar direct associations between dietary acidity and BP or hypertension risk in cross-sectional [10,13] and prospective [11,12] analyses in different age groups. In two prospective studies in older adults with a mean baseline age of either 65 or 70 years, however, no consistent associations with hypertension incidence were seen for different markers of dietary acid load [14,15]. The higher mean age in the study populations of these two studies may be one possible reason for the divergent findings, since BP seems to level off or even decreases in this age group [34]. This may indicate that BP is less responsive to environmental influences such as dietary acid load at a higher age. Additionally, other predictors of hypertension such as chronic kidney disease or arterial stiffness may become more important in older individuals.

In addition to observational studies on dietary acid load and BP, there is also experimental evidence for a link between acid–base status and BP from animal studies, showing that disturbances in acid–base balance with lower systemic pH may precede the development of hypertension [21,35]. A recent experimental study in humans also indicated that alkalinization may have an independent influence on BP: In the cross-over study of Conen et al. [36], a clear BP-decrease was observed among overweight, middle-aged individuals after administration of alkalizing potassium citrate, whereas BP was not influenced by potassium chloride. A similarly designed study also comparing the BP-effects of potassium citrate and potassium chloride did however not confirm these results [37]. The reasons for these conflicting findings are unclear, but differences in study duration, potassium citrate dose, and characteristics of the study populations such as antihypertensive medication use, especially of drugs influencing the potassium homeostasis, may have influenced the results.

Apart from the observed PRAL–BP associations, the present study also confirmed inverse associations of potassium intake with systolic and diastolic BP as well as hypertension prevalence in the DEGS1-population. Regarding the strength of these associations and the comparison with the PRAL–BP relation, our adjusted models (Table 2) indicated that a 10-mmol higher estimated 24-h potassium excretion (corresponding to 0.4 g higher potassium intake) would result in a 0.3 mmHg lower systolic BP. According to our regression analyses, a similar systolic BP reduction would result from a PRAL reduction of about 6 mEq/day, broadly corresponding to 150 g higher vegetable intake or a 70 g lower meat intake. When considering the FFQ-based potassium intake estimate, a higher difference of about 0.8 g would be needed for a similar reduction in systolic BP. These comparisons indicate that in our analyses, the BP-association was stronger for the urinary than for the dietary estimates of potassium intake. However, when interpreting these predictions, it has to be kept in mind that our adjusted models explained only 10% to 20% of the BP variance, indicating a high interindividual variation due to unknown influencing factors. A possible reason for the difference between urinary and dietary estimates could be that the semi-quantitative FFQ used in DEGS1 may not allow for sufficiently detailed intake estimates to clearly separate the BP-effects of potassium from other possibly correlated but counteracting nutrients. Whether similar problems exist for the FFQ-based PRAL estimate cannot be determined in the present study due to missing urinary markers of diet-dependent acid load such as renal net acid excretion or 24-h urine pH. In general, the reported associations in the present analysis are only of moderate strength, which is largely due to the cross-sectional design (and the respective high inter-individual variation) of the DEGS1 study. With respect to potassium excretion, a recent large observational study in more than 100,000 adults reported a very similar decrease in systolic BP of 0.75 mmHg per each 1 g higher potassium excretion [38].

Another point that needs discussion is that usually, a low PRAL diet is accompanied by a high potassium intake. Correspondingly, we found a significant inverse correlation of PRAL with both urinary biomarker- and FFQ-based estimated potassium intakes in the DEGS1 study population (data not shown). At least parts of the postulated PRAL effects on blood pressure could thus be potassium effects. Potassium intake has its own direct lowering effect on blood pressure and several mechanisms are discussed for this including a lowered sympathetic activity, an altered baroreceptor activity, and a reduced renin production as well as an increase of renal natriuresis [6]. An additional postulated mechanism for BP-reduction with higher potassium intake is the vasodilating effect of this mineral [5]. Since vascular tone is the main determinant of diastolic BP [39], this mechanism may account at least partly for the differential associations of PRAL and potassium with diastolic and systolic BP observed in the present study.

Apart from the potassium-related effects, a variety of mechanisms have been proposed that suggest a potassium-independent influence of PRAL on blood pressure. The strong buffering of blood pH usually prevents clear changes in circulating free protons after marked increases in dietary acid loads, but this broadly constant pH level is not without a price. Increased glucocorticoid secretion is needed to facilitate ammoniagenesis, which in turn ensures renal elimination of excess H^+^ via NH_4_^+^ [40,41], thus preventing stronger blood pH reductions. In line with this it has been shown that reduction of dietary acid load by administration of alkali salts reduces glucocorticoid secretion in healthy nondiabetic [42,43] and pre-diabetic subjects [36]. Concurrently, elevated cortisol levels such as in subclinical hypercortisolism are frequently related to hypertension [44]. Higher levels of serum uric acid (UA) may also partly explain a direct association of diet-dependent acid load with BP since higher UA levels are related to an increased hypertension risk [45] and reduction of the dietary acid load has been shown to increase renal UA excretion and reduce serum UA in healthy young females [46].

**Figure 2:**
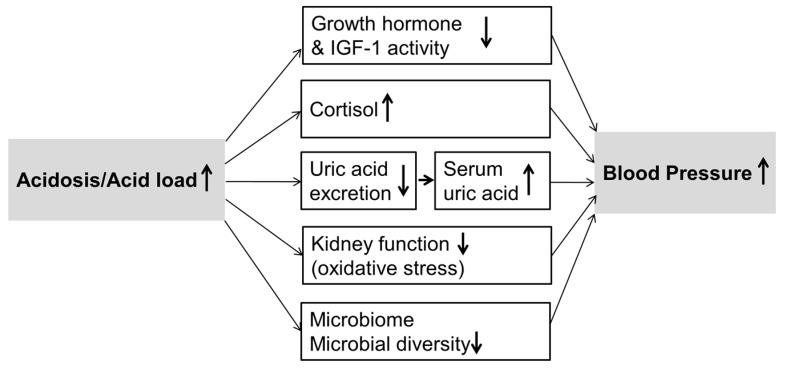
Discussed mechanisms involved in blood pressure changes due to an altered acid–base status of which particularly a potentially altered microbiome requires further experimental confirmation. IGF-1, insulin-like growth factor-1.

Moreover, also the influence of dietary acid load on gut microbiota and kidney function may mediate parts of the acid–base effects on blood pressure. The probable mechanisms are schematically represented in Figure 2. In comparison to western diets with high acid loads, more alkaline diets rich in fruits and vegetables and rich in dietary fibers result in a different microbiome [47] which may be more favorable with respect to BP [48]. Regarding the association of dietary acid load with kidney function, several studies in recent years have suggested that a lower dietary acid load may contribute to a reduced incidence [49] as well as slower progression [50] of chronic kidney disease. It has been suggested that a higher acid load may be detrimental to renal health due to prolonged high intra-renal ammonia concentrations [51]. Because a reduced kidney function may be related to higher BP-values already within the normal GFR-range, renal function represents a plausible link between dietary acid load and BP. In our analyses, however, adjustment for eGFR did not attenuate the observed associations of PRAL with systolic BP and hypertension prevalence. Moreover, our results were very similar after excluding participants with physician-diagnosed kidney impairment, microalbuminuria, or an eGFR < 60 mL/min/1.73 m^2^ (see Figure 1). This is somewhat in contrast to the recent analyses of Akter et al. [13], in which the association between dietary acid load and hypertension prevalence became non-significant after adjustment for eGFR.

Compared to some other studies examining the association between PRAL and BP outcomes [10,12,13], the median PRAL of −3.4 mEq/day in the DEGS1-population seems rather low. However, comparable [14] as well as much lower [52] PRAL levels in other populations have also been reported. Whether these large differences in average PRAL values truly reflect the variance in daily proton load or are partly attributable to different dietary assessment methods or partly incomplete capturing of dietary intake with FFQ is currently unclear. Differences with respect to higher PRAL values observed in healthy children [12] are at least partly explainable by coffee consumption since coffee has a negative (alkaline) PRAL of −1.7 mEq/100 g, and mean coffee intake in the DEGS1-population was almost 500 g/day, whereas it is negligible in most children.

Besides the potential benefit of a reduced PRAL with respect to BP, a lower dietary acid load has also been associated with a reduced incidence [49] and progression [50] of chronic kidney diseases as well as with a lower insulin resistance [53] and a lower diabetes risk [9]. At least theoretically, these different aspects could in combination contribute to a lowered cardiovascular risk with a more alkaline diet, but this hypothesis needs further investigation.

Several limitations of the present analysis need to be considered: First of all, the cross-sectional design of the DEGS1 study does not allow inferring a causal relationship between dietary acid load and BP. As has been mentioned above, the semiquantitative FFQ used in DEGS1 constitutes a further limitation. For the estimation of daily excretion of urinary sodium and potassium only spot urine samples were available. However, 24-h urine sampling is usually not feasible in large population-based studies and it has already been shown that the method of estimating 24-h excretion rates from spot urine mineral-creatinine ratios provides reasonable results within the DEGS1-population [54]. Additionally, as has been addressed above, it has to be considered that potassium intake and the PRAL estimates are interrelated and no definitive separation on their BP-relevance can be obtained in an observational study. However, PRAL potentially reflects a broader dietary pattern compared to potassium intake alone. Differences between these dietary predictors are also supported by our findings of a more robust association of PRAL with systolic BP and hypertension prevalence, whereas potassium intake might be more relevant for diastolic BP. Strengths of the present analyses include detailed and standardized questionnaires and examinations in DEGS1 allowing for control of a large number of potential confounders of the investigated diet-BP associations. Moreover, to our knowledge, this is the first time that the relevance of dietary acid load for BP has been directly compared to the established BP-association of potassium in a large representative population sample.

## 5. Conclusions

We have demonstrated that a higher dietary acid load is significantly associated with higher systolic BP and a higher hypertension prevalence in the general adult population living in Germany, and that these findings are independent of BMI, sodium intake, kidney function, relevant medication, and further BP-influencing factors. Albeit of only moderate strength, the PRAL–BP associations seem to be comparable to those observed for potassium intake and support available evidence for a more alkaline dietary pattern for BP reduction. Besides, the findings of the present analyses indicate that biomarker information from spot urine samples may supplement FFQ-based estimates in epidemiological studies on diet–disease relations.

## Figures and Tables

**Table 1:** Characteristics of the German Health-Interview and Examination Survey for Adults (DEGS1) study population in sex-balanced quintiles of dietary potential renal acid load (PRAL) (*n* = 6788).

Median PRAL, mEq/Day	−30.8 (−44.3, −23.7)	−12.7 (−16.9, −7.9)	−4.5 (−7.7, 0.4)	3.9 (−0.6, 7.8)	15.5 (9.3, 23.2)	*p*
*n*	1356	1358	1358	1358	1358	
Women, %	49.4 (46.2, 52.7)	51.2 (47.9, 54.5)	52.4 (48.9, 55.9)	49.7 (46.1, 53.4)	49.4 (46.1, 52.7)	0.7
Age, years ^a^	49.9 (48.9, 50.9)	52.5 (51.3, 53.7)	50.1 (49.0, 51.3)	45.7 (44.6, 46.8)	40.4 (39.4, 41.3)	<0.0001
BMI, kg/m^2 a^	27.0 (26.6, 27.3)	26.9 (26.6, 27.3)	27.0 (26.6, 27.3)	26.9 (26.6, 27.3)	26.4 (26.0, 26.8)	0.1
Systolic BP, mmHg ^a^	123.8 (122.8, 124.7)	124.7 (123.5, 125.9)	124.6 (123.5, 125.6)	123.9 (122.9, 124.9)	123.7 (122.7, 124.7)	0.5
Diastolic BP, mmHg ^a^	73.4 (72.7, 74.0)	73.5 (72.8, 74.2)	73.5 (72.9, 74.1)	73.1 (72.5, 73.7)	72.9 (72.2, 73.6)	0.7
Hypertension prevalence ^b^, %	31.8 (28.8, 34.8)	36.3 (32.7, 40.0)	34.9 (31.7, 38.1)	32.2 (28.7, 35.7)	24.6 (22.0, 27.3)	<0.0001
Diuretic use, %	4.4 (3.3, 5.5)	5.6 (4.1, 7.1)	5.4 (3.8, 7.0)	5.4 (3.8, 6.9)	2.4 (1.6, 3.1)	0.001
Beta blocker use, %	15.1 (12.9, 17.3)	17.8 (15.2, 20.4)	15.3 (12.8, 17.8)	14.5 (11.9, 17.0)	9.4 (7.8, 11.0)	<0.0001
Total cholesterol, mg/dL ^a^	203.6 (200.6, 206.6)	207.7 (204.4, 210.9)	205.1 (201.3, 209.0)	201.6 (198.5, 204.7)	196.2 (193.2, 199.2)	<0.0001
Estimated GFR ^c^, mL/min/1.73 m^2 a^	93.3 (91.5, 95.1)	89.6 (87.8, 91.5)	92.5 (90.5, 94.4)	93.6 (91.8, 95.4)	99.0 (96.9, 101.1)	<0.0001
Smoking						
Daily or occasionally, %	32.6 (29.2, 36.0)	22.8 (19.9, 25.8)	25.9 (23.0, 28.9)	30.7 (27.3, 34.0)	34.0 (30.6, 37.4)	<0.0001
Former smoker, %	27.3 (24.4, 30.2)	32.0 (28.9, 35.0)	32.1 (29.3, 35.0)	27.8 (24.7, 31.0)	23.2 (20.4, 26.1)	
Never smoker, %	40.1 (36.6, 43.6)	45.2 (42.0, 48.4)	41.9 (38.6, 45.2)	41.5 (38.0, 45.0)	42.7 (39.4, 46.1)	
Sports activity						
No sports activity, %	32.3 (28.8, 35.8)	31.8 (28.2, 35.3)	31.3 (27.8, 34.8)	32.4 (28.9, 36.0)	34.8 (31.7, 38.0)	0.2
<2 h per week, %	38.8 (35.3, 42.4)	43.5 (40.1, 46.9)	42.6 (39.2, 46.0)	44.2 (40.8, 47.6)	40.4 (37.3, 43.5)	
>2 h per week, %	28.9 (25.3, 32.4)	24.8 (21.9, 27.7)	26.1 (22.8, 29.4)	23.4 (20.3, 26.6)	24.8 (21.9, 27.7)	
Socioeconomic Status (SES)						
Low	18.4 (15.7, 21.0)	17.1 (14.1, 20.1)	18.9 (15.9, 22.0)	16.3 (13.5, 19.1)	22.8 (20.0, 25.7)	<0.0001
Medium	62.0 (58.5, 65.5)	58.4 (54.7, 62.0)	58.9 (55.5, 62.4)	62.6 (59.3, 66.0)	61.9 (59.1, 64.6)	
High	19.6 (16.8, 22.4)	24.5 (21.3, 27.7)	22.2 (19.0, 25.3)	21.1 (18.3, 23.9)	15.3 (13.0, 17.6)	
Alcohol						
0 g/day, %	15.7 (13.2, 18.2)	11.3 (8.7, 13.9)	16.4 (13.7, 19.1)	13.2 (11.0, 15.5)	16.3 (13.5, 19.0)	0.04
<10/20 g/day, %	67.9 (64.8, 71.1)	73.8 (70.8, 76.9)	67.0 (63.7, 70.3)	71.6 (68.5, 74.6)	67.6 (64.6, 70.5)	
>10/20 g/day, %	16.4 (13.6, 19.1)	14.9 (12.5, 17.2)	16.6 (14.1, 19.0)	15.2 (13.0, 17.5)	16.2 (14.1, 18.3)	
Estimated urinary Na-Excretion, mmol/day ^d^	161.1 (103.2, 242.5)	156.0 (97.1, 232.4)	157.1 (102.5, 227.2)	160.7 (98.9, 232.9)	165.1 (103.9, 236.2)	0.2
Estimated salt intake, g/day ^d^	9.4 (6.0, 14.2)	9.1 (5.7, 13.6)	9.2 (6.0, 13.3)	9.4 (5.8, 13.6)	9.6 (6.1, 13.8)	0.2
Estimated K-Excretion, mmol/day ^d^	93.1 (67.9, 121.9)	84.9 (64.5, 112.2)	85.2 (59.9, 113.8)	78.9 (60.4, 106.0)	73.9 (54.2, 102.1)	<0.0001
Estimated K-Intake, mg/day ^d^	4403 (3540, 5664)	3120 (2595, 3785)	2700 (2185, 3407)	2619 (1975, 3207)	2793 (2196, 3606)	<0.0001
Meat consumption ^e^, g/day ^d^	66.4 (37.9, 103.2)	66.2 (40.0, 97.8)	67.6 (42.6, 97.8)	81.3 (49.2, 117.9)	118.0 (78.2, 183.3)	<0.0001
Milk product consumption ^f^, g/day ^d^	314.6 (142.9, 616.3)	262.1 (130.8, 457.6)	242.2 (127.1, 428.9)	242.6 (128.5, 425.6)	245.6 (127.9, 457.3)	<0.0001
Fruit and vegetable consumption, g/day ^d^	461.1 (232.4, 827.9)	357.7 (213.5, 530.7)	269.8 (166.1, 420.5)	211.6 (128.1, 330.3)	179.3 (97.2, 303.8)	<0.0001

^a^ Data presented as mean (95% Confidence interval); ^b^ Hypertension was defined as BP values ≥ 140/90 mmHg or antihypertensive medication use in physician-diagnosed hypertension; ^c^ Glomerular filtration rate, calculated according to the four-variable Modification of Diet in Renal Disease (MDRD) formula; ^d^ Data presented as median (Q1, Q3); ^e^ Consumption of meat including poultry, ham, and sausages; ^f^ Consumption of milk, cream cheese, cheese, curd cheese, soured milk and yoghurt; BMI, body mass index; BP, Blood pressure.

**Table 2:** Multiple linear regression analyses on the association of PRAL and potassium in sex-balanced quintiles as continuous predictor variables with blood pressure levels as continuous outcome variables in the total DEGS1 study sample (*n* = 6788).

		Total Sample (*n* = 6788)		
Predictor	Outcome	β (95% CI)	*P*_trend_	*R*^2^
	**Systolic blood pressure**			
PRAL (FFQ), mEq/day	Basic model ^a^	0.0486 (0.0216, 0.0756)	**0.0005** ^c^	0.1570
	Adjusted model ^b^	0.0521 (0.0250, 0.0792)	**0.0002** ^c^	0.1927
K-Intake (FFQ), g/day	Basic model ^a^	−0.3327 (−0.7114, 0.0461)	0.08	0.1551
	Adjusted model ^b^	−0.3969 (−0.7734, −0.0204)	**0.04** ^c^	0.1906
K-Excretion, mmol/day	Basic model ^a^	−0.0119 (−0.0235, −0.0003)	**0.04** ^c^	0.1551
	Adjusted model ^b^	−0.0330 (−0.0455, −0.0205)	**<0.0001** ^c^	0.1944
	**Diastolic blood pressure**			
PRAL (FFQ), mEq/day	Basic model ^a^	0.0119 (−0.0070, 0.0308)	0.2	0.1032
	Adjusted model ^b^	0.0148 (−0.0038, 0.0334)	0.1	0.1481
K-Intake (FFQ), g/day	Basic model ^a^	−0.1727 (−0.4088, 0.0634)	0.2	0.1033
	Adjusted model ^b^	−0.2546 (−0.4891, −0.0202)	**0.03** ^c^	0.1484
K-Excretion, mmol/day	Basic model ^a^	−0.0069 (−0.0141, 0.0004)	0.06	0.1034
	Adjusted model ^b^	−0.0154 (−0.0236, −0.0071)	**0.0003** ^c^	0.1500

^a^ Adjusted for age, sex, and BMI; ^b^ Basic model, additionally adjusted for size of blood pressure cuff, fasting duration (> or <8 h), smoking status, natrium excretion, alcohol intake, diuretics, beta-blockers, eGFR, serum glucose, and total cholesterol. ^c^ Bold numbers indicate significant *p*-values (<0.05); PRAL, potential renal acid load; DEGS1, German Health-Interview and Examination Survey for Adults; CI, confidence interval; FFQ, food frequency questionnaire; BMI, body mass index; eGFR, estimated glomerular filtration rate calculated according to the four-variable MDRD formula.

**Table 3:** Linear regression analyses on the association of PRAL and potassium in sex-balanced quintiles as continuous predictor variables with blood pressure levels as continuous outcome variables in a reduced DEGS1 study sample of participants without antihypertensive medication (*n* = 4677).

		Sample without Antihypertensive Medication (*n* = 4677)		
Predictor	Outcome	β (95% CI)	*P*_trend_	*R*^2^
	**Systolic blood pressure**			
PRAL (FFQ), mEq/day	Basic model ^a^	0.0375 (0.0094, 0.0657)	**0.009** ^c^	0.2059
	Adjusted model ^b^	0.0375 (0.0086, 0.0664)	**0.01** ^c^	0.2385
K-Intake (FFQ), g/day	Basic model ^a^	−0.2648 (−0.685, 0.1553)	0.2	0.2045
	Adjusted model ^b^	−0.2210 (−0.6379, 0.1960)	0.3	0.2370
K-Excretion, mmol/day	Basic model ^a^	−0.0128 (−0.0257, 0.0002)	0.05	0.2050
	Adjusted model ^b^	−0.0280 (−0.0420, −0.0140)	**0.0001** ^c^	0.2406
	**Diastolic blood pressure**			
PRAL (FFQ), mEq/day	Basic model ^a^	0.0068 (−0.0119, 0.0256)	0.5	0.1878
	Adjusted model ^b^	0.0064 (−0.0129, 0.0258)	0.5	0.2132
K-Intake (FFQ), g/day	Basic model ^a^	−0.1954 (−0.4718, 0.0810)	0.2	0.1882
	Adjusted model ^b^	−0.1880 (−0.4597, 0.0836)	0.2	0.2136
K-Excretion, mmol/day	Basic model ^a^	−0.0085 (−0.0167, −0.0002)	**0.04** ^c^	0.1886
	Adjusted model ^b^	−0.0144 (−0.0238, −0.0049)	**0.003** ^c^	0.2154

^a^ Adjusted for age, sex, and BMI; ^b^ Basic model, additionally adjusted for size of BP cuff, fasting duration (> or <8 h), smoking status, natrium excretion, alcohol intake, eGFR, serum glucose, and total cholesterol; ^c^ Bold numbers indicate significant *p*-values (<0.05); PRAL, potential renal acid load; DEGS1, German Health-Interview and Examination Survey for Adults; CI, confidence interval; FFQ, food frequency questionnaire; BMI, body mass index; eGFR, estimated glomerular filtration rate calculated according to the four-variable MDRD formula.
